# Protocol for the ECLIPSE-BRAZIL: a prospective cohort study on biomarkers and risk stratification for Preeclampsia

**DOI:** 10.1186/s12884-026-09253-4

**Published:** 2026-07-02

**Authors:** Ana Luísa Torres Tavares, Vanessa Duarte Pasa, Jesiel Francisco de Jesus Fernandes Martins Lima, Júlia Zanon Pereira, Mara Quintela Maia, Giovanni Paulo Diniz, Aislander Junio da Silva, Flávia Ribeiro de Oliveira, Kelly Cristina Anatólio Do Carmo, Priscila Arcebispo Léo, Séphora Augusta Cardoso Queiroz, Suzana Maria Pires Do Rio, Melina Barros-Pinheiro, Danyelle Romana Alves Rios, Leila Cristine Do Nascimento Melo, Valéria Cristina Sandrim, Mariana Bertozzi Matheus, Luiza-Oliveira Perucci, Luci Maria Sant’Ana Dusse, Lara Carvalho Godoi, Patrícia Nessralla Alpoim

**Affiliations:** 1 Department of Clinical and Toxicological Analysis, School of Pharmacy, Federal University of Minas Gerais (UFMG), Belo Horizonte, Minas Gerais Brazil; 2 Department of Gynecology and Obstetrics, School of Medicine, Federal University of Minas Gerais (UFMG), Belo Horizonte, Minas Gerais Brazil; 3 Blood Transfusion Service, Odete Valadares Maternity Hospital, Hospital Foundation of the State of Minas Gerais (FHEMIG), Belo Horizonte, Minas Gerais Brazil; 4 Odete Valadares Maternity Hospital, High-Risk Prenatal Outpatient Clinic, Hospital Foundation of the State of Minas Gerais, Belo Horizonte (FHEMIG), Minas Gerais, Brazil; 5 Graduate Program in Health Sciences, Dona Lindu Midwest Campus, Federal University of São João del-Rei (UFSJ), Divinópolis, Minas Gerais Brazil; 6 Clinical Hematology Laboratory, Dona Lindu Midwest Campus, Federal University of São João del-Rei (UFSJ), Divinópolis, Minas Gerais Brazil; 7 Department of Biophysics and Pharmacology, Institute of Biosciences (IBB), São Paulo State University (UNESP), Botucatu, São Paulo, Brazil; 8 Graduate Program in Biomolecular and Pharmacological Sciences, Institute of Biosciences (IBB), São Paulo State University (UNESP), Botucatu, São Paulo Brazil; 9 Department of Obstetrics, Gynecology and Reproductive Sciences, University of California San Diego (UCSD), San Diego, CA United States of America; 10 Clinical Pathology Sector, Technical College (COLTEC), Federal University of Minas Gerais (UFMG), Belo Horizonte, Minas Gerais Brazil

**Keywords:** Preeclampsia, Longitudinal Study, Prospective Cohort, Biomarkers, Predictive Factors, Maternal Health

## Abstract

**Background:**

Preeclampsia (PE) is a leading cause of maternal and perinatal morbimortality, particularly in low- and middle-income countries. Despite advances in clinical management, its etiology and pathogenesis remain unclear, and effective predictive biomarkers are lacking. The Brazilian Longitudinal Study for the Investigation of Preeclampsia (ECLIPSE-BRAZIL) is a prospective cohort designed to investigate genetic, immunological, hemostatic, biochemical, and angiogenic profiles in pregnant women at risk for PE, aiming to identify biomarkers that effectively and affordably predict PE risk early in pregnancy.

**Methods:**

ECLIPSE-BRAZIL follows 500 to 1,000 pregnant women receiving care at the High-Risk Prenatal Care clinics in Belo Horizonte and Divinópolis, Minas Gerais, Brazil. Clinical and laboratory assessments are conducted at four gestational stages and postpartum for PE cases. Clinical data, including sociodemographic, clinical and behavioral factors, and blood and urine samples are systematically collected. Plasma and urine levels of inflammatory markers, angiogenic factors, oxidative stress parameters, extracellular vesicles, microRNAs, endothelial and platelet markers will be analyzed. Participants are stratified by PE development and gestational age at diagnosis. Standardized criteria and data protocols ensure rigor, while quality check and audits enhance data reliability. To minimize follow-up loss, participants are contacted frequently, with flexible sampling and the use of hospital records.

**Discussion:**

ECLIPSE-BRAZIL will provide novel insights into PE pathophysiology, improving disease prediction and management. By studying a diverse population, we will identify biological signatures to inform health innovations: development of cost-effective diagnostic tools, point-of-care tests and simple clinical algorithms to identify women at risk before symptoms become severe. Finally, we will provide a deeper understanding of biological mechanisms driving PE and its subtypes, including endothelial function and immune response, which may lead to the discovery of new therapeutic targets. ECLIPSE-BRAZIL represents a major advancement in PE research, integrating a comprehensive evaluation of clinical, biochemical, and molecular markers throughout pregnancy. Its findings may inform public health policies and improve clinical practices worldwide.

**Trial registration:**

Not applicable. This is an observational cohort study approved by the Research Ethics Committees, as documented under the following CAAE numbers: 12471918.0.0000.5149, 12471918.0.3002.5119, 12471918.0.3003.5130, 12471918.0.3006.5545.

**Supplementary Information:**

The online version contains supplementary material available at https://doi.org/10.1186/s12884-026-09253-4.

## Background

Maternal health is a human right that remains neglected in many parts of the world, as highlighted by alarming statistics. Each year, over half a million women die from pregnancy-related causes, with 99% of these deaths occurring in low-income and developing countries. In these countries, pregnancy and childbirth complications are the leading causes of death among women of reproductive age, despite most of these deaths being preventable. Socioeconomic inequalities often restrict access to quality healthcare, perpetuating these avoidable tragedies [1]. Within this context, PE stands out as one of the major obstetric syndromes, contributing significantly to maternal and perinatal morbidity and mortality [2].

PE is characterized by the onset of hypertension accompanied by proteinuria and/or target organ dysfunction after the 20th week of gestation. It affects 2% to 8% of pregnancies and can result in severe outcomes [1], such as eclampsia (generalized seizures not attributed to other causes) and HELLP syndrome (hemolysis, elevated liver enzymes, and low platelet count) [3]. Additionally, PE complications include cerebrovascular accidents, liver rupture, pulmonary edema, and acute renal failure, all of which can be fatal [4]. Preterm delivery and intrauterine growth restriction (IUGR) are common adverse perinatal outcomes associated with PE, with fetal death occurring in severe cases [5]. Furthermore, growing evidence suggests that women with a history of gestational hypertension or PE face an increased lifetime risk of developing cardiovascular and metabolic diseases, a risk also observed in their offspring [1, 3].

The etiology of PE is complex and multifactorial, giving rise to numerous hypotheses to explain the interplay of risk factors and clinical manifestations [3]. The prevailing theory suggests that PE originates from abnormal placentation, which triggers an excessive release of antiangiogenic molecules, such as soluble fms-like tyrosine kinase-1 (sFlt-1) and soluble endoglin (sEng), along with other factors such as pro-inflammatory markers and reactive oxygen species. These factors, historically referred to as “toxins” (hence the earlier term “toxemia of pregnancy”), when present in high levels, induce endothelial dysfunction, vasoconstriction, and immune dysregulation, adversely affecting maternal organ systems and the fetus [6]. A dysfunctional placenta, rather than the fetus itself, is required for the development of PE, as evidenced by its occurrence in cases such as hydatidiform moles [7]. Notably, the only definitive treatment for PE is delivery with the complete removal of the placenta [8].

Current clinical management during pregnancy focuses on proven preventive strategies for PE. These include the use of low-dose aspirin for pregnant women at high risk, calcium supplementation, antihypertensive medicines to manage severe hypertension, and magnesium sulfate to prevent and control eclamptic seizures [9].

While a better understanding of the etiology of PE is essential, its multifactorial nature makes prevention challenging. Therefore, there should be a continuous effort to identify early biomarkers to screen pregnant women at risk of developing PE, which would enable better management of these patients and ultimately reduce complications associated with this clinical condition [9]. Some international guidelines suggest the use of biomarkers to improve PE diagnosis, particularly the sFlt-1/PlGF ratio, especially in developed countries, as recommended by the American College of Obstetricians and Gynecologists (ACOG). In Brazil, all country`s health regulatory agency ANVISA-approved products to determine sFlt-1 and PlGF levels are imported, leading to external technology dependence, as well as high costs and delays in delivery.

Within this context, the Longitudinal Study For The Investigation of Preeclampsia (ECLIPSE-BRAZIL) (in Portuguese, “Estudo Longitudinal para Investigação da Pré-Eclâmpsia”) arises as a prospective cohort aimed to characterize the genetic, immunological, hemostatic, biochemical, and angiogenic profiles throughout pregnancy in women with risk factors for developing PE, who are receiving prenatal care at health institutions in the state of Minas Gerais, Brazil. In the cohort, we seek to identify novel PE biomarkers, as well as determine the predictive efficiency of existing ones (sFlt-1 and PlGF) within the study population. We also intend to develop cost-effective diagnostic tests, point-of-care technologies, and clinical algorithms to predict the risk of PE and diagnose it before symptoms become severe. Furthermore, we aspire to understand the biological mechanisms driving the disease and its subtypes so that new interventions can be effectively tailored to the underlying etiology.

## Methods

### Study design

ECLIPSE-BRAZIL is a prospective cohort study that follows 500–1,000 pregnant women throughout their pregnancy, a number defined to ensure statistically reliable and representative results, considering the incidence of PE in the population and the feasibility of recruitment within the available resources over the 10-year study period [10]. Launched in November 2021, it initially involved pregnant women receiving care at the High-Risk Prenatal Care clinic at Odete Valadares Maternity Hospital in Belo Horizonte, Brazil, through a collaboration between researchers from the Federal University of Minas Gerais and the municipal health system (CAAE: 12471918.0.0000.5149 / 12471918.0.3002.5119) [10]. Recently, the study expanded to Divinópolis, Minas Gerais, Brazil, through a new partnership with researchers from the Dona Lindu Midwest Campus of Federal University of São João del-Rei (CAAE: 12471918.0.3003.5130 / 12471918.0.3006.5545) [10]. This partnership has enabled the inclusion of new participants from the municipality, with prenatal care provided at the Center for Medical Specialties of the Polyclinic, as well as at the High-Risk Prenatal Care Service of a medium-sized hospital in the city [10].

### Study population: selection and invitation

The selection of pregnant women invited to participate in the study is conducted by trained graduate students of UFMG and UFSJ, considering specific inclusion and exclusion criteria. Inclusion criteria are based on the ACOG recommendations and consider the presence of one strong risk factor or two moderate risk factors for developing PE. Strong risk factors include a history of PE in a previous pregnancy, a family history of PE in first-degree relatives, paternal family history of PE, chronic hypertension, pregestational diabetes, and chronic kidney disease. Moderate risk factors include nulliparity, extremes of maternal age at the time of first pregnancy (younger than 18 or older than 35 years), multifetal gestations, non-Caucasian ethnicity, a long interpregnancy interval (over 10 years), antiphospholipid antibody syndrome, and body mass index (BMI) exceeding 30 kg/m². A summary of these risk factors is provided in Table 1.

**Table 1:** Inclusion criteria outlining strong and moderate risk factors for the development of PE

Inclusion Criteria	
Strong risk factors	PE in a previous pregnancy
	Family history of PE in first-degree relatives
	Paternal family history of PE
	Chronic hypertension
	Pregestational Diabetes
	Chronic kidney disease
Moderate risk factors	Nulliparity
	Extremes of maternal age at the time of first pregnancy (younger than 18 or older than 35 years)
	Multifetal gestations
	Non-Caucasian ethnicity
	Long interpregnancy interval (over 10 years)
	Antiphospholipid antibody syndrome
	BMI exceeding 30 kg/m²

Hypertension in pregnancy is defined as systolic blood pressure (BP) ≥ 140 mmHg and/or diastolic BP ≥ 90 mm Hg on two separate measurements taken four hours apart at rest. In PE, hypertension arises in the context of increased systemic vascular resistance and afterload, along with decreased cardiac output and intravascular volume compared to normotensive pregnant controls [11].

The exclusion criteria include comorbidities such as coagulation disorders, cardiovascular diseases, autoimmune diseases, liver diseases, cancer, and any form of bleeding.

In addition to the inclusion and exclusion criteria, pregnant women eligible for participation are selected based on a gestational age of 12 to 19 weeks, which marks the first collection interval of the longitudinal study.

Eligible participants are invited to join the study on the day of their prenatal consultation. Upon arrival at the health unit, they receive an invitation to participate along with a general explanation about the study. If they agree to participate, they are asked to sign the Informed Consent Form. For participants under 18 years old, the same procedure is followed in the presence of a legal guardian. Once consent is obtained, data collection and biological sample collection procedures are initiated.

### Collection of clinical data

Data collection is carried out systematically and standardized to ensure consistency. Once the pregnant woman agrees to participate, she undergoes a structured interview covering personal information, medical history, dietary habits, and psychological and emotional factors affecting her health and well-being. The questionnaire detailed in Table 2 constitutes the patient’s clinical record for this study, which is also attached in Additional file 1.

**Table 2:** Questionnaire forming the patient’s clinical record

Patient’s clinical record	
Personal information	Name Date of Birth Age Ethnicity Marital Status Nationality / Place of Birth Education Level Address Phone / WhatsApp number
Medical history	Comorbidities COVID-19 history and vaccination record Pregnancy details: Type of pregnancy (single or multiple), baby’s sex, number of previous pregnancies, history of preterm birth, miscarriage, interpregnancy interval, whether pregnancies were with the same partner Primary complaints Presence of risk factors for PE Height Blood Type
Dietary habits and consumption frequency	Intake of soda, alcohol, fish and seafood, milk and dairy products, nuts (including peanuts, hazelnuts, and walnuts), whole grains, tea, water Food allergies
Psychological and emotional aspects	Was the pregnancy planned? Do you have a support network? Employment status and working hours If you have children, who takes care of them while you work? Are you in a committed relationship with the baby’s father? How would you describe your relationship with the baby’s father and his support during pregnancy? How do you perceive yourself in relation to this pregnancy?

The clinical record is securely archived within the hospital in a designated research cabinet and accompanies all biological sample collections conducted at specific intervals. Meanwhile, clinical data recorded by gynecologists during prenatal consultations are retrieved from the hospital’s information system. This system is available to all students involved in the project, ensuring that relevant updates are documented in each participant’s clinical record. Table 3 outlines the clinical data collected at each sampling moment throughout the gestational period.

**Table 3:** Clinical data collected at each sampling moment throughout the gestational period

Patient’s clinical record	
Clinical data	Symptoms
	Blood pressure
	Weight
	BMI
	Presence of edema
	Proteinuria
	Medications

Additionally, after delivery, data regarding the procedure, including the delivery date and any reported complications, are documented, along with information about the newborn. This information is summarized in Table 4.

**Table 4:** Clinical data related to delivery and the newborn

Patient’s clinical record	
Delivery	Date
	Gestational Age
	Delivery type: cesarean or vaginal
	Reason for delivery: induced or natural
	If diagnosed with PE, gestational age at diagnosis
	Need for hospitalization
Newborn	Singleton or multiple newborn
	Sex
	Weight
	Size
	Intrauterine Growth Restriction (IUGR)
	APGAR scores at 1 and 5 min
	Need for support
	Need for hospitalization

At conclusion of data collection, clinical records are digitized, with the names of the pregnant women recorded in acronym form. The data is then encoded and protected by password access, ensuring that only the researchers involved in the project can view the information. These steps are implemented to maintain the privacy and security of the participants, in full compliance with the Brazilian General Data Protection Law.

The simultaneous collection of clinical data and biological samples is essential for understanding the relationship between clinical factors and biological changes in PE. This integrated approach helps uncover the underlying mechanisms of PE, improving its diagnosis and treatment strategies, and ultimately enhancing maternal and fetal outcomes through effective gestational monitoring.

### Collection of biological samples

Biological samples are collected alongside clinical data during four distinct phases of pregnancy. For patients who develop PE, a fifth phase is carried out during the puerperium, up to 42 days postpartum. The dates for meeting with study participants during each phase of pregnancy are determined in advance based on a review of the physicians’ appointment schedules. Table 5 outlines the timeline for the different collection phases.

**Table 5:** Clinical data and biological sample collection phases

Collection phases					
	1º phase	2º phase	3º phase	4º phase	5º phase
Gestational period	12–19 weeks	20–29 weeks	30–34 weeks	> 34 weeks	Puerperium, up to 42 days postpartum
Patients	All pregnant women enrolled in the study				Only pregnant women who developed PE

In each phase, blood samples are collected by students involved in the project, who have received proper training to perform all necessary procedures, or by a hospital phlebotomist. The necessary materials for blood collection are provided by the project coordinators. Blood is drawn into five tubes: one serum tube (no anticoagulant added), two tubes containing the anticoagulant citrate, one tube containing heparin, and one EDTA-containing tube. Each tube has a 4 mL capacity, totaling 20 mL of blood per collection. Each type of collection tube enables the analysis of specific blood parameters. The Serum tube will be used for biochemical analyses and heparin for extracellular vesicles assessment. One of the citrate tubes is specifically used to obtain platelet-poor plasma (PPP), which will be utilized exclusively for the thrombin generation assay (TGA) [12]. Immediately after collection, a complete blood count (CBC) is performed using the Pentra XLR (Horiba) equipment, with the EDTA tube, which is ideal for preserving blood cell integrity and ensuring accurate hematological assessment.

In addition to blood collection, a urine sample is also obtained from the participants. An appropriate collection tube is provided, and the responsible student gives instructions on how to properly collect the sample to ensure it is representative. The urine is then analyzed using a biochemical strip (Sensitive – Cral^®^), which evaluates several important parameters: ketones, glucose, protein, specific gravity, pH, nitrites, blood, bilirubin, urobilinogen, and leukocytes. The detection of protein in the urine, known as proteinuria, is a crucial indicator of PE [3]. This analysis is also important for identifying signs of urinary tract infections or other imbalances, offering valuable insights into renal health and the overall condition of the participant. In certain cases, the results may be shared with the medical team overseeing the patient’s care. Urine collection can also be utilized for future analyses, enhancing the depth of the research.

After completing all initial collections and procedures in the hospital’s blood bank and biochemistry laboratory, the blood and urine samples are processed following the standard operating procedure (SOP) established by the project coordinators. Initially, the samples are centrifuged using the Sorvall™ ST 16 centrifuge (Thermo Fisher Scientific), with the following settings: citrate tubes are centrifuged at 3000 g for 15 min, other blood tubes at 4000 rpm for 10 min, and the urine sample at 1500 g for 10 min. To obtain the PPP, an additional centrifugation step is performed. After the first centrifugation, 1500 µL of plasma is carefully pipetted and transferred to a microcentrifuge tube, which is then centrifuged again under the same conditions.

After centrifugation, the samples are aliquoted using a pipette into standardized storage tubes (cryotubes). Each tube is properly labeled with the participant’s code (“GE + sequential number”), the participant’s name, the origin tube, and the collection period, which is identified by predefined color codes: red for the first collection, blue for the second, green for the third, and yellow for the fourth collection. The fifth postpartum collection is not color-coded. The aliquots are prepared as follows: 500 µL of plasma or serum are stored in each of the sixteen tubes; 1200 µL of PPP in one tube; the red blood cell concentrate from the sediment in the EDTA tube is stored in one tube; and 2000 µL of urine in each of the two tubes. In total, 20 cryotubes are obtained and placed in designated plastic containers in a nitrogen tank at − 196 °C for preservation within the hospital.

When the nitrogen tank reaches full capacity, the logistics for transporting the samples are managed by responsible personnel. The samples are then transferred to an − 80 °C ultrafreezer (Coldlab) located in the biochemistry laboratory at the Department of Clinical and Toxicological Analysis at the UFMG. The samples collected in Divinópolis are transferred to an − 80 °C ultrafreezer (Thermo Fisher Scientific) located in the Dona Lindu Midwest Campus of UFSJ. Once stored in the ultrafreezer, the samples remain available for analysis as needed by the students and coordinators involved in the project.

### Group stratification

Stratification into groups is a key component of the ECLIPSE-BRAZIL study, enabling a comprehensive analysis of the differences between pregnant women who develop PE and those who do not. This classification is made by physicians of the National Risk Attention Program Brazil, who consider specific clinical and laboratory criteria for the diagnosis of PE, such as blood pressure levels, proteinuria, and other relevant findings that help assess potential complications.

In terms of blood pressure, PE is diagnosed when systolic BP reaches 140 mmHg or higher, and/or diastolic BP reaches 90 mmHg or higher, based on two measurements taken at least four hours apart, after the 20th week of gestation in a woman with previously normal blood pressure. Severe hypertension, defined as systolic BP of 160 mmHg or higher or diastolic BP of 110 mmHg or higher, can be confirmed within a short interval (minutes), enabling the prompt initiation of antihypertensive therapy [13].

In terms of proteinuria, PE is diagnosed when there is 300 mg or more of protein in a 24-hour urine collection (or an equivalent amount from a timed collection), a protein/creatinine ratio greater than 0.3 mg/dL, or a 2 + reagent strip reading (used only when other quantitative methods are unavailable). However, in the absence of proteinuria, PE can still be diagnosed if hypertension is accompanied by at least one of the following complications: thrombocytopenia (platelet count less than 100,000/µL), renal insufficiency (serum creatinine levels greater than 1.1 mg/dL or doubling of baseline creatinine levels), liver dysfunction (elevated transaminases at least twice the normal levels), pulmonary edema, neurological symptoms, including persistent headache unresponsive to medications and unexplained by alternative diagnoses, or visual disturbances [13].

In addition to these criteria, physicians also document the gestational age at which PE is diagnosed. This condition can be further subdivided into two phenotypes: early onset (EOPE), diagnosed before 34 weeks of gestation, and late onset (LOPE), diagnosed at or after 34 weeks [14].

Comparing pregnant women with and without PE according to their gestational ages allows for a deeper understanding of the biological, clinical, and molecular differences between them. This approach helps isolate key variables that aid in identifying specific patterns and biomarkers of PE and its various clinical manifestations. Such insights are crucial for improving diagnostic accuracy and developing personalized management strategies for patients with PE, ultimately advancing gynecological and obstetric care.

### Bias control and follow-up strategies

To ensure the validity and reliability of the study findings, several measures are implemented to minimize selection, information, and follow-up biases.

Selection bias is addressed by rigorously applying standardized eligibility criteria across all recruitment sites, ensuring a uniform selection process. The research team receives standardized training to consistently apply these criteria at all study centers. Additionally, periodic assessments are conducted to monitor the distribution of participants across key characteristics such as age, ethnicity, and risk factors, ensuring comparability between recruitment sites. To further reduce selection bias, all cases of non-enrollment are systematically documented, allowing the research team to identify potential patterns that could affect the study’s generalizability.

To minimize information bias, standardized data collection protocols are established across all study sites. Clinical assessments, sample collection, and questionnaire administration follow uniform procedures, reducing variability in data acquisition. All personnel involved in data collection receive extensive training to ensure methodological consistency. Furthermore, the same medical devices and laboratory assays are used across all study centers, preventing measurement discrepancies. To maintain data quality, periodic audits are conducted to detect inconsistencies and ensure adherence to standardized protocols.

In addition to these strategies, specific measures are implemented to prevent loss to follow-up, a critical factor in maintaining the study’s integrity. A participant is only classified as a definitive loss after multiple unsuccessful contact attempts. Temporary absences, such as missing a scheduled visit but returning later, are not considered definitive losses. To enhance retention, regular follow-up is conducted via WhatsApp messages and phone calls, reinforcing the importance of continued participation and minimizing dropout rates. In cases where participants cannot be reached, their medical records from participating health institutions are reviewed to obtain relevant clinical information and determine their health status.

By implementing these strategies, the study aims to reduce potential sources of bias, ensure methodological rigor, and enhance the completeness and reliability of follow-up data.

### Analysis of laboratory markers

This study aims to evaluate polymorphisms and mutations in genes associated with the synthesis of inflammatory mediators, enzymes directly or indirectly linked to the nitric oxide pathway, and genes involved in hemostasis and coagulation in the blood cells of pregnant women. Additionally, it seeks to determine the plasma PE postpartum levels of pro-inflammatory and regulatory cytokines, proteins involved in the resolution of inflammation, Brain-Derived Neurotrophic Factor (BDNF), coagulation markers, fibrinolysis components, markers of endothelial injury, natural anticoagulants, and angiogenesis markers.

This study also aims to assess the plasma levels of liver function markers, including Aspartate Aminotransferase (AST), Alanine Aminotransferase (ALT), and gamma-Glutamyl Transferase (gamma-GT); kidney function markers such as urea and creatinine; and oxidative stress markers, including Thiobarbituric Acid-reactive Substances (TBARS) and (3-(4,5-Dimethylthiazol-2-yl)-2,5-Diphenyltetrazolium Bromide (MTT). Additionally, the study will measure levels of Asymmetric Dimethylarginine (ADMA), cyclic Guanosine Monophosphate (cGMP), uric acid, and glucose.

Furthermore, the study will assess the circulating platelet counts, along with platelet parameters, including mean platelet volume (MPV), platelet distribution width (PDW), and plateletcrit (PCT). It will also evaluate platelet-monocyte aggregates (PMA) and platelet-neutrophil aggregates (PNA). An investigation into the association of ABO-Rh blood groups and PE will also be conducted.

Regarding immunological characterization, serum samples will be used to quantify chemokines, pro-inflammatory cytokines, regulatory cytokines and growth factors (CXCL8, CCL11, CCL3, CCL4, CCL2, CCL5, CXCL10, IL-1β, IL-6, TNF, IL-12, IFN-γ, IL- 17 A, IL-1Ra, IL-4, IL-9, IL-10, IL-13, PDGF, VEGF, G-CSF and IL-7) using the Bio-Plex Pro Human Cytokine Assay. In the context of PE, it is crucial to understand the immune and inflammatory processes involved in the development and progression of the disease. Dysfunction in the balance between these cytokines may contribute to the maintenance of a chronic inflammatory state, aggravating PE. Additionally, the quantification of proteins involved in inflammation resolution, such as C-Reactive Protein (CRP), may provide insights into the body’s ability to control and resolve inflammatory processes. The analysis of these biomarkers could be incorporated into routine prenatal laboratory tests, enabling early identification, risk stratification, and monitoring of therapeutic efficacy. Furthermore, with a detailed understanding of the immune response in PE, more specific therapies could be developed, targeting inflammation modulation through regulation or stimulation of cytokines, for example. This could open pathways for innovative treatments aimed not only at symptom control but also at reversing the inflammatory processes that contribute to disease progression.

Placenta-derived extracellular vesicles (EV), necessary in maternal–fetal communication, might contribute to PE pathogenesis, and the elevation of EV has been described in this condition. PE-EV contain phosphatidylserine in their surface, resulting in blood clot formation, fibrin depositions, and contributing to a PE hypercoagulability state. In addition, PE-EV exhibit an increase of tissue factor, and is involved in the activation of monocytes, macrophages, and the vascular endothelium. Therefore, characterization of circulating EVs through flow cytometry has great potential in clinical practice. Thus, we intend to evaluate the following markers in the membrane of EV: trophoblast, CD49e, VCAM-1, ICAM-1, VWF, CD62P, CD41b, CD42a, CD51/CD61, CD69, CD142, and CD14. As biomarkers, EVs can be analyzed to identify specific patterns that indicate the onset of the disease or its severity, which would enable the development of rapid and non-invasive diagnostic kits, such as blood tests, to monitor pregnant women at risk of PE, assisting in the clinical management of the disease. Furthermore, EVs can act as vehicles for delivering targeted therapies to specific cells, allowing for more precise treatments, and can be used as therapeutic targets to modulate inflammatory responses, angiogenic processes, and other pathological pathways associated with PE and related conditions. Additionally, collecting data on vesicles throughout pregnancy makes it possible to create predictive models that help identify pregnant women at higher risk of developing PE, providing effective screening and preventive interventions.

In relation to epigenetic analyses, plasmatic microRNAs regulating the expression of genes involved in placentation will be identified, which can be used as biomarkers. MicroRNA extraction from plasma will be performed using the NucleoSpin^®^ purification and isolation kit (Macherey-Nagel), followed by real-time PCR analysis with SYBR^®^ Green (Thermo Fisher Scientific) on the QuantStudio^®^ system (Applied Biosystems – Thermo Fisher Scientific) for precise quantification. The expression of these microRNAs will be compared between patients who developed and those who did not develop PE, as well as in relation to clinical parameters. The results obtained will be processed and analyzed through bioinformatics tools, allowing for an in-depth interpretation of the data.

It is important to note that the quantification of the sFlt-1/PlGF ratio, a valuable marker for the diagnosis of PE, is not widely performed in developing countries like Brazil due to the high cost of imported kits for measuring these markers. Brazil remains entirely dependent on external technology, as all sFlt-1 and PlGF-related diagnostic products registered with ANVISA are of foreign origin. To change this scenario, we aim to develop national Enzyme-Linked Immunosorbent Assay (ELISA) kits to quantify sFlt-1 and PlGF in blood and urine samples, making testing more accessible. Moreover, to ensure that the diagnosis can be carried out in locations with lack of resources and advanced equipment, we plan to develop a rapid lateral flow test for PE. The samples collected in the ECLIPSE cohort will be used in the diagnostic kits standardization, allowing for the calibration of the tests to align with the characteristics of Brazil’s population.

Additionally, we propose the development and validation of a point-of-care thrombin generation assay (TGA). It is known that PE is associated with fibrin deposition in microvasculature, which results in compromised placental perfusion, intrauterine fetal growth restriction and maternal organs dysfunction. Indeed, the process of intravascular coagulation activation may explain some of the PE clinical features and may be more relevant to fetal outcome than blood pressure. Thrombin has pro and anticoagulant properties and is considered the main protein involved in hemostasis regulation. The thrombin generation assay is a method that evaluates the capacity of thrombin generation in plasma ex vivo. Unlike other hemostasis monitoring methods available routinely in laboratories, in which fibrin clot occurs when less than 5% of total thrombin has been generated, the TGA can evaluate hemostasis in an overall manner with greater sensitivity. In this regard, the rapid TGA can be used to understand PE hypercoagulability state and screen severe PE outcomes. Undoubtedly, this product would come to fill an extremely important gap, considering the frequency and severity of hemostatic changes and disseminated intravascular coagulation disorders in PE.

In addition, the research group led by Professor Dr. Valéria Cristina Sandrim of the Biological Institute of São Paulo State University (UNESP) in Botucatu, which focuses on the identification of biomarkers, therapeutic targets, and potential treatments for PE, also collaborates with the ECLIPSE project. The group employs an in vitro model of endothelial cells incubated with plasma from pregnant women with PE to quantify nitric oxide (NO), reactive oxygen species (ROS), nitrite, and cell viability, aiming to assess the impact of maternal plasma on endothelial function and to evaluate the potential benefits of pharmacological agents, drugs, and natural compounds in mitigating these effects [15–19].

Considering that the release of harmful factors into maternal circulation is crucial for the development of endothelial dysfunction and the clinical symptoms of PE [6], incubating endothelial cells with the plasma of pregnant women at different gestational ages can provide valuable insights into endothelial function [15, 20, 21]. To assess NO production, intracellular NO levels will be quantified using Diaminofluorescein-FM diacetate (DAF-FM), while nitrite, a stable product of NO, will be measured in the cell culture supernatant using the Griess assay. Furthermore, comparing the effects of plasma from women who developed PE with those who remained healthy during pregnancy will enhance our understanding of the impact of these circulating factors on the endothelium [22].

Finally, we aim to develop a Machine Learning (ML) model based on sociodemographic, clinical variables, family history, laboratory, and vital signs data for PE. ML techniques have emerged as a valuable tool for the diagnosis and prognosis of multiple diseases, given that they can expose non-linear relationships between biomarkers, revealing patterns not immediately apparent. The high-performing ML model for PE detection will be constructed with ECLIPSE study parameters and will use a novel synthetic data generation method, DAS, to enhance dataset quality. It will introduce a simpler, interpretable ridge regression model, which will identify links between laboratorial parameters and PE. We believe that this novel application of ML has the potential to improve PE monitoring protocols, making them more efficient and broadly accessible.

### Statistical analysis

Data analysis will be conducted using the SPSS software version 26. Normality will be assessed with the Shapiro-Wilk test. Comparisons between two groups will be performed using the Student’s T-test for parametric variables, while ANOVA will be applied for comparisons involving three or more groups. Non-parametric variables will be compared using the Mann-Whitney test for two-groups comparisons or Kruskal-Wallis test for three or more groups. Correlations between the parameters will be evaluated using Pearson’s correlation for quantitative variables and Spearman’s correlation for qualitative variables. A P-value < 0.05 will be considered statistically significant.

### Quality control

In ECLIPSE-BRAZIL, data and results quality are ensured through a rigorous control process. Pilot studies are first conducted to identify and rectify potential flaws in instruments and data collection procedures, ensuring a well-structured methodology. The team responsible for sample collection and clinical examinations undergoes continuous training aligned with established protocols, which are readily accessible and periodically reviewed.

The reliability of the instruments used is verified through rigorous quality control measures at both the hospitals and universities involved in the study. This ensures that all measurements are accurate and consistent. Furthermore, data is meticulously monitored, with any identified errors promptly corrected. Regular monthly meetings provide a platform for continuous improvement, while the team undergoes additional training as needed to maintain precision and efficiency in all processes.

This set of measures, overseen by the project coordinators, ensures that ECLIPSE-BRAZIL upholds the highest quality standards, enabling the generated data to make a strong and reliable contribution to advancing the understanding of PE.

### Data and results management

In ECLIPSE-BRAZIL, data and results management is carefully overseen by the coordinating faculty, who closely monitor data collection and analysis through text message groups and monthly meetings. This continuous monitoring ensures adherence to established protocols and allows for swift adjustments whenever necessary.

All data collected through clinical forms is digitized and securely stored in a password-protected cloud storage repository, with access restricted to students involved in the project. This ensures data security and compliance with the General Data Protection Law. Additionally, blood tests results are stored alongside the data to ensure traceability and preserve information integrity.

This data management process safeguards all information while ensuring its availability for analysis. By upholding rigorous quality standards, the ECLIPSE-BRAZIL project ensures the generation of reliable results, strengthening research efforts to deepen the understanding of PE.

### Notification of study results and participant referral

Participants` contact information is securely stored to facilitate future communication, including delivery of a brief report summarizing the research results in which their samples were utilized.

Critical findings from routine tests, such as complete blood counts or urine analysis via biochemical strips, are promptly reported to the responsible medical team for further evaluation and necessary intervention. This proactive approach enhances clinical follow-up, contributing to improved outcomes in the context of PE.

Additionally, any results with significant implications for maternal and infant health will be communicated to the relevant institutions and authorities to support public health initiatives and policymaking.

## Discussion

ECLIPSE-BRAZIL has the potential to generate valuable insights into the early identification of PE onset, a condition with high prevalence and significant impact on maternal and infant health. This is particularly crucial in developing countries like Brazil, where the burden of gestational diseases remains a major public health concern.

As a longitudinal study following a prospective cohort of 500 to 1,000 pregnant women throughout gestational, ECLIPSE-BRAZIL represents pioneering research that addresses a critical gap in the literature of PE. By establishing a clear timeline, the study provides valuable insight into the temporality of factors associated with PE, a key element in determining causality. Specifically, examining the evolution of biomarkers and clinical characteristics throughout pregnancy is crucial for investigating maternal factors contributing to the development of PE. Such studies are rare in the literature, partially due to the evolving diagnostic criteria of PE and high cost [10]. Additionally, collecting biological samples from the same cohort of women over the course of pregnancy presents challenges, particularly due to participant attrition and follow-up losses, which we plan to address through flexible sample collection, considering accessible hospital locations and timing suited to the patient’s health and well-being; team training to provide personalized support, address concerns, and emphasize the study’s importance; and active monitoring to detect signs of attrition and strengthen adherence to both the project and prenatal care in collaboration with the medical team.

ECLIPSE-BRAZIL stands out for studying PE in a heterogeneous population with known risk factors, enabling the identification of disease patterns and biomarkers. The collection of multiple biological samples, such as blood and urine, at different stages of pregnancy allows for a detailed analysis of clinical markers, such as cytokines, microRNAs, proteins associated with inflammation, coagulation, and oxidative stress, as well as parameters of hepatic, renal, and platelet function and circulating microparticles. Furthermore, the samples obtained in this cohort will allow the development and standardization of national and cost-effective diagnostic tools, such as sFlt-1 and PlGF quantification ELISAs, point-of-care tests and clinical algorithms. By comparing pregnant women with and without PE, this study deepens the understanding of the syndrome and its associated risk factors, promoting early and accurate detection, and the determination of effective strategies for preventing, diagnosing, monitoring, and treating patients.

In summary, ECLIPSE-BRAZIL will provide groundbreaking research into the development and diagnosis of PE, with its findings poised to make a significant impact on maternal-fetal health. The study results will be particularly valuable for Brazil and other low-income countries, advancing both local and global healthcare efforts.

## Supplementary Information

Additional file 1. pdf – Clinical Record: archive containing questionnaire answers and clinical data related to each patient, which is used for the follow-up throughout the longitudinal study.

## Data Availability

No datasets were generated or analysed during the current study.

## Abbreviations

ACOG: American College of Obstetricians and Gynecologists
ADMA: Asymmetric Dimethylarginine
ALT: Alanine Aminotransferase
ANOVA: Analysis of Variance
ANVISA: National Health Surveillance Agency (Brazil)
APGAR: Appearance, Pulse, Grimace, Activity, and Respiration (Newborn Health Score)
AST: Aspartate Aminotransferase
BDNF: Brain-Derived Neurotrophic Factor
BMI: Body Mass Index
BP: Blood Pressure
CAAE: Certificate of Ethical Appreciation Presentation (Brazil)
CBC: Complete Blood Count
cGMP: Cyclic Guanosine Monophosphate
CNPq: National Council for Scientific and Technological Development (Brazil)
COLTEC: Technical College of UFMG (Federal University of Minas Gerais)
CRP: C-Reactive Protein
DAF-FM: Diaminofluorescein-FM diacetate
ECLIPSE: Longitudinal Study for Investigation of Preeclampsia
ELISA: Enzyme-Linked Immunosorbent Assay
EOPE: Early Onset Preeclampsia
EV: Extracellular Vesicles
FAPEMIG: Research Support Foundation of the State of Minas Gerais
FHEMIG: Hospital Foundation of the State of Minas Gerais
GAMA-GT: gamma-Glutamyltransferase
IUGR: Intrauterine Growth Restriction
LOPE: Late Onset Preeclampsia
ML: Machine Learning
MPV: Mean Platelet Volume
MTT: (3-(4,5-Dimethylthiazol-2-yl)-2,5-Diphenyltetrazolium Bromide
NO: Nitric Oxide
PCR: Polymerase Chain Reaction
PCT: Plateletcrit
PDW: Platelet Distribution Width
PE: Preeclampsia
PE-EV: Preeclampsia and Extracellular Vesicles
PlGF: Placental Growth Factor
PMA: Platelet-Monocyte Aggregates
PNA: Platelet-Neutrophil Aggregates
PPP: Platelet-Poor Plasma
ROS: Reactive Oxygen Species
sENG: Soluble Endoglin
sFlt-1: Soluble Fms-like Tyrosine Kinase-1
SPSS: Statistical Package for the Social Sciences Software
TBARS: Thiobarbituric Acid Reactive Substances
TGA: Thrombin Generation Assay
UCSD: University of California, San Diego
UFMG: Federal University of Minas Gerais
UFOP: Federal University of Ouro Preto
UFSJ: Federal University of São João del-Rei
UNESP: São Paulo State University
