# Supplementary material for: Protocol for the ECLIPSE-BRAZIL: a prospective cohort study on biomarkers and risk stratification for Preeclampsia
Source: BMC Pregnancy Childbirth. 2026 Jul 2;26:1043. doi: 10.1186/s12884-026-09253-4 (PMC13595709; doi:10.1186/s12884-026-09253-4)
Supplement: Supplementary file 1 [file 12884_2026_9253_MOESM1_ESM.pdf]

CLINICAL RECORD

ECLIPSE-BRAZIL: Longitudinal Study For The Investigation of Preeclampsia

**Patient ID:**	**Medical record:**	**Group: ☐ Non-PE ☐ PE**

**1. Personal information:**
Name:
Date of birth:
Age:
Ethnicity:
Marital status:
Nationality / Place of birth:
Education Level:
Address:
Phone / WhatsApp number:

**2. Comorbidities / Important notes:**
Autoimmune, liver, kidney, cardiovascular diseases; diabetes; cancer; complications in previous pregnancy<br><br><br><br>Had COVID-19? When?<br>Vaccinated? How many doses?

**3. Information on pregnancy(ies)**		
Multiple pregnancy? ☐ Yes ☐ No		Sex: ☐ Boy ☐ Girl ☐ Both
PDA (Pregnancy / Delivery / Abortion): / /		
First pregnancy? ☐ Yes ☐ No		
Same father? ☐ Yes ☐ No		
Previous deliveries:	vaginal ______	cesarean section______
Interpregnancy interval:		
Any preterm birth? ☐ Yes ☐ No		
Main complaints:<br>☐ Headache ☐ Epigastric pain ☐ Scotoma ☐ Others:		
Risk factors for PE:		
☐ Previous PE	☐ Extreme age range: ≤18 - ≥35 years	
☐ Family history of PE	☐ Long interpregnancy interval: >10 years	
☐ Paternal family history of PE	☐ Multiple pregnancy	
☐ Primigravida	☐ Blood type AB	
☐ Primipaternity	☐ Non-white ethnicity	
☐ Chronic kidney disease (CKD)	☐ Diabetes mellitus	
☐ Chronic arterial hypertension (CAH)	☐ BMI ≥35 kg/m²	

4. Clinical information
Height:
Blood type:

	12-19 weeks	20-29 weeks	30-34 weeks	>34 weeks
Date				
Gestational age				
Symptoms				
Blood pressure				
Weight				
BMI				
Edema	☐ Yes ☐ No	☐ Yes ☐ No	☐ Yes ☐ No	☐ Yes ☐ No
Proteinuria	☐ Yes ☐ No	☐ Yes ☐ No	☐ Yes ☐ No	☐ Yes ☐ No
Medications	☐ Nifedipine<br>☐ Methyldopa<br>☐ Calcium carbonate<br>☐ Ferrous sulfate<br>☐ Aspirin (ASA)<br>☐ Others	☐ Nifedipine<br>☐ Methyldopa<br>☐ Calcium carbonate<br>☐ Ferrous sulfate<br>☐ Aspirin (ASA)<br>☐ Others	☐ Nifedipine<br>☐ Methyldopa<br>☐ Calcium carbonate<br>☐ Ferrous sulfate<br>☐ Aspirin (ASA)<br>☐ Others	☐ Nifedipine<br>☐ Methyldopa<br>☐ Calcium carbonate<br>☐ Ferrous sulfate<br>☐ Aspirin (ASA)<br>☐ Others

5. Urine collection

	12 – 19 w	20 – 29 w	30 – 34 w	> 34 w	Postpartum
	__ : __ h	__ : __ h	__ : __ h	__ : __ h	__ : __ h
Ketone					
Glucose					
Protein					
Density					
pH					
Nitrite					
Blood					
Bilirubin					
Urobilinogen					
Leukocytes					

6. Intake		
Was the pregnancy planned? ☐ Yes ☐ No		
Support network? ☐ Yes ☐ No		
Do you work outside the home? ☐ Yes ☐ No	Workload:	Activity:
If you have other children, where/with whom do they stay when you work?<br>☐ Daycare ☐ School ☐ Grandparents ☐ Baby's father ☐ Other:		
Are you in a serious relationship with the baby's father? ☐ Yes ☐ No		
How is your relationship with the baby's father? ☐ Bad ☐ Normal ☐ Good		
Do you feel supported by your partner / the baby's father? ☐ None ☐ Little ☐ A lot		
If you could describe how you feel about this pregnancy in one word, what would it be?<br>☐ Sad ☐ Happy ☐ Normal ☐ Other:		
Observations:		

7. Nutrition	
Do you consume soda? ☐ Yes ☐ No	Amount:
Have you consumed alcoholic beverages? ☐ Yes ☐ No	Amount:
Do you consume fish and seafood? ☐ Yes ☐ No<br>Which ones?	Amount:
Do you consume milk and dairy products? ☐ Yes ☐ No	Amount:
Do you consume nuts (peanuts, hazelnuts, walnuts)? ☐ Yes ☐ No	Amount:
Do you consume whole foods? ☐ Yes ☐ No<br>Which ones?	Amount:
Do you drink any kind of tea? ☐ Yes ☐ No	Amount:
Daily water intake: ☐ < 1L ☐ 1L – 2L ☐ > 2L	
Do you have any food allergies? ☐ Yes ☐ No<br>Which ones?	
Observations:	

8. Delivery		
Date:	GA:	Delivery type: ☐ Vaginal ☐ Cesarean section
Reason:		
PE diagnosis: ☐ Yes GA______ ☐ No		Hospitalization needed: ☐ Yes ☐ No
Observations:		

9. Newborn			
Multiple pregnancy? ☐ Yes ☐ No		Sex: ☐ Boy ☐ Girl ☐ Both	
Weight:	Size:	IUGR: ☐ Yes__ ☐ No	APGAR: 1st _ / 5st _
Need for support: ☐ Yes ☐ No		Need for hospitalization: ☐ Yes ☐ No	
Observations:			

10. Important information
